# Observations on Irritable Bladder; with Cases

**Published:** 1816-07

**Authors:** Robert Arrowsmith

**Affiliations:** Surgeon


### *Observations on irritable Bladder; with Cases.*

By Robert Arrowsmith, Surgeon.

Among the many calamitous disorders to which the human fabric is exposed, there is scarcely any with which I am acquainted, that produces such a degree of silent and unheeded suffering, as the affection on which I propose to comment; and whatever may be suggested in elucidation of the origin and nature of the disorder, or towards the alleviation of that suffering, demands the serious consideration of every practitioner.

Symptoms of irritation of the bladder are very frequent among females; but it is the aggravated stage of the symptoms, when they are liable to be confounded with stone, that I shall now notice, and, according to my observation, they are not confined to either the single or married state. I will here take the liberty of quoting a passage from Mr. John Burns "Principles of Midwifery." He observes, (page 63) that "in many cases the symptoms of stone are met with though none can be found in the bladder. This is most frequently the case with young girls previous to the establishment of the catamenia, or with women of an irritable habit. There is no organic disease; nor have I ever known it in such people end in a diseased structure of the bladder or kidneys; indeed, they rarely complain of uneasiness about the kidneys. I have tried many remedies, such as soda, uva ursi, narcotics, antispasmodics, tonics, and the warm and cold bath; but cannot promise certain relief from any of these. In process of time, the disease subsides and disappears. The use of a bougie may be of service." He then refers to a note on this subject, and remarks, (page 589) that "in a case of this kind, described by Mr. Patton, as a spasmodic affection of the neck of the bladder, calomel appeared to cure the complaint."

I have said that irritation of the bladder is a common source of complaint in females, but in the advanced or aggravated state of the complaint, the suffering is very severe. Mrs. E. 45 years of age, came under my care in April 1815, for this complaint, with which she had been attacked seven or eight months before. It is remarkable that the first attack was momentary. She was engaged in an employment peculiarly apt to disorder the constitution, the lustreing of porcelain; in which process a gaseous substance, disengaged by the action of nitro-muriatic acid on platina or gold, is constantly inhaled. She was one afternoon seized with violent pain in the loins, with sickness and faintness. She had previously been tolerably well; but from that time, the signs of irritation of the bladder commenced, and she was seldom a day without a paroxysm of pain. The first attack strikingly resembled the passage of a calculus along the ureter; but nothing of the kind was ever observed in the urine, and she had no repetition of the attack. When she first consulted me, she complained of debility, and had lost flesh; her appetite was impaired, the tongue furred, the bowels irregular, but generally constipated; she was thirsty, and occasionally sick. The pain in the bladder occurred daily; it apparently came on when the bladder became distended to a certain point, and the urine was then discharged by repeated contractions of the detrusor urinæ, which might be almost called involuntary: they were continued till the bladder was emptied and for sometime afterwards, and shortly the pain became less severe, and then altogether ceased. The urine was generally natural in its obvious and sensible properties, but from her own description a considerable quantity of mucus had been discharged; and in the commencement, some albumen. Indeed, when I first saw her, the presence of the latter substance was indicated by the action of alcohol and a solution of the muriate of quicksilver.

I ordered her some aperient medicines; which were at that time necessary; and she was afterwards directed to take the nitrate of potass with powdered gum acacia. The alkali, although taken only in the dose of ten grains twice a day, was productive of a great increase of the local irritation, and of a severe attack of fever. The paroxysms of pain occurred with great severity twice in the day; they were moderated by the warm bath, and occasionally by tepid local bathing. Purgation with oleum ricini and manna, the employment of antimonials and diluents, were the means of removing the severe accession of fever. When it had subsided, the local irritation existed with unabated force, and two or three times specks of blood were noticed in the urine. I know not whether increase of pain after the bladder has emptied itself is characteristic of urinary calculus, but certainly it did not exist in the present instance; and this circumstance partly induced me to doubt the existence of stone. I sounded my patient, and no stone could be discovered. The meatus urinarius seemed inflamed, and the urethra was unusually sensible in its whole course.

Of the various means subsequently employed in this case, I am compelled in general to speak unfavourably. Although from what has been said of the uva ursi, some benefit might have been anticipated from its use, yet in this instance it was perfectly inert. Very small doses of the subcarbonate of potass afforded some benefit, but of a very temporary nature. It was imagined that its inefficacy might be ascribed to the system becoming accustomed to its operation; but subsequent experience proved that its powers were not augmented by increasing the dose, however gradually this were done; indeed, a contrary result was obtained. Balsam. copaibæ and narcotics were not useful. It seemed in vain to expect permanent good from means employed to mitigate the local inflammation simply, and the plan was consequently abandoned. The tone of the stomach continued impaired, and the functions of the bowels irregularly performed. She was still thirsty, and the skin was dry; but the pulse was not accelerated, and she menstruated regularly. The improvement of the general health was attempted by mild purgatives and bitters; and under this plan she speedily became better. In three weeks she was decidedly relieved. The bladder quietly retained more urine, and the attacks of pain were less frequent, and less severe. The purgatives that were found to answer best were aloes, rhubarb, and ipecacuanha in combination. She continued to take them for three months; and from their operation she gradually, but perfectly, recovered of her complaint. She discontinued her employment from the commencement of my attendance, and still continues well.

Mrs. P. aged 64, consulted me on the 10th of June, 1815, on account of pain in the bladder; with which she had been affected some weeks. She described it as occurring in paroxysms, during one of which I first saw her. Her suffering was exceedingly great. Opium, oleum ricini, and the warm bath, afforded her temporary, but imperfect relief; the paroxysms of pain, however, recurred, and frequently to that degree, as to require a repetition of these means. The cause of the complaint was not obvious. There was nothing in the nature of her occupation that appeared conducive to the symptoms under which she laboured. The pain came on at irregular intervals, accompanied by a desire to expel the contents of the bladder. Mucus, and specks of blood occasionally passed, but these were infrequent occurrences. The pain continued for some time after the urine was expelled, but was certainly not increased. The introduction of the sound demonstrated the non-existence of stone. Her pulse was quick, the skin hot and dry, the tongue dry, but not furred; she had nausea, and a total disinclination to food. The bowels acted about every other day, and the biliary secretion was natural. She took antimonials and the sulphate of potass; afterwards, subcarbonate of potass, balsam. copaibæ, uva ursi, and other remedies of the same kind; but without obtaining the desired relief. She then tried the mild purgative plan; under which, a rapid amendment took place. The purgative medicine was composed of aloes, rhubarb, and antimonial powder. She continued them for a month, and has since remained well.

About three years ago, the following case came under my observation. An unmarried lady, 22 years of age, had suffered for some years with "vaginal discharge," accompanied by considerable irritation of the bladder. I have frequently observed a similar combination of symptoms, in which the vaginal discharge has preceded the irritation of the bladder, and the latter affection I have ascribed to the communication of the morbid action to the mucous membrane of the urethra and bladder, from continuous sympathy. Her general health was much disordered. The tongue was much furred; the appetite impaired; and she had lost flesh and strength. The bowels were irregular; and the catemenia appeared every three weeks. Pain in the right side was complained of occasionally, and there was slight yellowness of the conjunctiva. Her habits were sedentary; and there existed in her constitution a great susceptibility of impression from causes either mental or physical, but from the former more particularly. The affection of the bladder commenced with sensations of uneasiness; but in the progress of the complaint it manifested itself more decidedly, and eventually became a source of painful, but silent misery. It was accompanied by the desire to void urine; and shortly afterwards subsided. From her own description, mucus unquestionably had been passed with the urine, but no blood was ever observed. The disease in the commencement received little attention from her medical adviser. It was treated as a case of leucorrhœa dependant on debility, and the usual mode of treatment under such circumstances by tonics, as myrrh, steel, soda, and stimulants, as olibanum and balsam. copaibæ, was followed. No relief was obtained by their employment, and subsequently other remedies were prescribed; seemingly with reference also to the state of the bladder. Lime water and uva ursi were for a considerable time persevered in, and pills of soap and aloes taken when the bowels were not sufficiently open. Relief was obtained from the operation of the pills, but they were not regularly continued; they were repeated occasionally only. The symptoms did not yield to the medicines employed. They varied certainly, yet such changes could not, with any probability, be ascribed to the remedies, but to the operation of the general causes of salubrity. The disease was perplexing: it was sometimes contemplated as serious in its nature; at others, regarded as the offspring of a clouded imagination. At the end of a year and a half, however, so far from being retarded, it had evidently become more serious. The vaginal discharge was unabated, and the irritability of the bladder had assumed a more formidable aspect. It was now accompanied by pain in the region of the kidnies, and increased sensibility of the urethra. The emaciation and debility had become more conspicuous. Stone was, at this period, thought of; but the removal of the debility by tonics was the most obvious indication in the judgment of the practitioner. Steel, under every variety of form, and other tonics, were persisted in with an obstinacy, which nothing but profound prejudice and insensibility to evidence could account for. Their inutility became apparent. The sound was introduced, but no calculus was discovered. It occurred to the practitioner, that the complaints *might* arise from local inflammatory action; and he therefore suggested the adoption of an "antiphlogistic" treatment: it was accordingly adopted. Rhubarb and sulphate of potass were given, so as to produce a decided purgative effect. The superiority of this mode of treatment over the tonic plan soon became apparent. It was found adviseable to diminish the medicine, so as to produce a more gentle but permanent effect. From a continuance of this plan, the symptoms subsided; and in six weeks, the irritability of the bladder and vaginal discharge were removed. The local remedies found useful in this case were tepid local bathing, and the vapour of warm water.

In a constitution so susceptible of disorder as I have stated this lady's to have been, it perhaps cannot be expected that she should have remained free from indisposition. As far as regards the affection of the bladder and the vaginal discharge, she has certainly suffered little, if at all. Since the termination of those sufferings, there has existed occasional derangement of the hepatic functions, and some obscure symptoms consequent thereon I presume; but such derangement has been much mitigated, or removed, by very small doses of blue pill.

A case analogous to the preceding has lately occurred to me. Perhaps, for severity and duration of suffering, this case exceeds those I have already recited. The subject is a lady, 26 years of age, and she has been afflicted with irritability of the bladder for several years. The degree of debility was not so great as in the preceding case, although, in many other material points, they corresponded. It should be remarked, that she had a severe attack of gastritis previously to the occurrence of the symptoms now the object of our attention. An experienced physician, who visited her, paid much attention to the state of the stomach, which was continually deranged. Purgatives, however, formed not a part of the plan of cure. The sound was introduced by a distinguished fashionable surgeon without detecting stone. He ordered her a chemical preparation, sold under the title of the "Sedlitz powder," to take every morning, and the hyosciamus at night. The powder operates mildly on the bowels; and since she began its use, she has enjoyed a considerable freedom from complaint. I have seen her only since she began the plan she now follows, and there seems to be decisive proofs of its success. At present, if she is without the "Sedlitz powder," she suffers uneasy sensations. Sulphate of magnesia has been substituted for it, but it produced much irritation. The hyosciamus was not continued.

The foregoing cases illustrate and confirm some of the observations of Mr. Burns; and the inefficacy of the articles of the Materia Medica, commonly resorted to in the treatment of irritable bladder, is thus pretty clearly ascertained. There needs no argument to prove the superiority of the purgative plan of treatment over that usually adopted; for, on whatever principles these remedies were prescribed, it is at least manifest, that their first and most evident operation was on the bowels, and that the irritability of the bladder ceased as a secondary effect. In the first, second, and fourth cases, I had no evidence of defective or morbid secretion of bile: in the third, there can be no doubt but the functions of the liver were improperly performed. In all, the stomach was deranged; and it is highly probable, the intestinal canal participated in the disorder. It seems a reasonable inference, I think, from what is known relative to the laws of sympathy, that the present disorder should be regarded as a sympathetic morbid action, consequent on the derangement of the stomach and bowels;—but I do not wish to insist upon it, as I choose rather to confine myself to practical points.

My experience authorizes me to say, that the combination of ipecacuanha, or a preparation of antimony, with the purgative, materially increases its efficacy.

I confess myself more disposed to censure than to praise the mode of investigation pursued in the present instance, in its application to medical science, as bordering too much on experiment, and as not being sufficiently analytical to afford indisputable evidence; yet, perhaps, as rough sketches of truth, which men of more acuteness and discrimination will more accurately define, the present cases deserve to be recorded.

It would have been gratifying to me, to offer something in elucidation of the diagnosis in this disease, because I consider it an imperative duty on the part of all men devoted to the improvement of medical science, to make it the first object of their study. The learned Professor of Anatomy and Surgery to the London College of Surgeons has, in the Medico-Chirurgical Transactions (vol vi. p. 261) countenanced opinions which would discourage us from pursuing our inquiries in this department of medicine. Every production of his pen, (distinguished as he is for profound and comprehensive acquirements) is entitled to the most respectful attention, and the opinions he has countenanced are, it must be admitted, true, as far as regards the organ, on the diseases of which he was treating; but not, I hope, in relation to pathological science in general: for it must be borne in mind, that it is a necessary consequence in this, as in physical science generally, that "in proportion to its progress, its enquiries become more minute and refined."

The Pathologic labours of Dr. Farre are well calculated to reflect lustre on the talents which produced them, and the science they are destined to improve; and I cannot but avail myself of the present opportunity of offering the humble tribute of my admiration to the sagacity and philosophic views disclosed in his works now in the course of publication.

In the cases I have detailed, there certainly was no increase of pain after the bladder had emptied itself; and if increase of pain after the bladder has expelled its contents be characteristic of the presence of urinary calculus, these cases stand in contrast with that diagnostic sign. It is a point of importance, but a farther observation of cases must decide the question.

Longton, Staffordshire,
May 17, 1816.

